# A Case of Brodifacoum-Induced Epiglottitis

**DOI:** 10.7759/cureus.47286

**Published:** 2023-10-18

**Authors:** William N Doyle, Kenneth Dumas, Justin K Arnold

**Affiliations:** 1 Emergency Medicine, University of South Florida Morsani College of Medicine, Tampa, USA

**Keywords:** vitamin-k therapy, international normalized ratio (inr), adulterated synthetic cannabinoids, brodifacoum, epiglottitis

## Abstract

This case report presents a 33-year-old woman who presented to the emergency department with abdominal pain and gingival and vaginal bleeding. She admitted to using synthetic cannabinoids, and contamination with brodifacoum was suspected, for which qualitative testing was positive. The patient was discharged with an improved international normalized ratio (INR) seven days later with oral vitamin K. Fourteen days after discharge, she re-presented with widespread ecchymosis, leg swelling, and intermittent gingival and vaginal bleeding. Her INR was again elevated. She was controlled with oral vitamin K therapy, stabilized, and discharged three days later. Twenty-eight days following the second discharge, the patient re-presented with oral swelling, right eye ecchymosis, and vaginal bleeding after abstaining from vitamin K therapy for two weeks. A bedside nasopharyngolaryngoscopy showed the base of the tongue, epiglottis, aryepiglottic (AE) folds, arytenoids, and false vocal folds were all edematous with ecchymosis. Due to the diffuse epiglottic and supraglottic edema, the patient was intubated to avoid further decompensation. After receiving IV and oral vitamin K, she was extubated two days later. Her INR fully normalized, and she was then discharged on day 4. Our case of epiglottitis could demonstrate thermal injury associated with smoking synthetic cannabinoids, but given diffuse ecchymosis and severe coagulopathy, hematoma associated with brodifacoum poisoning was considered the most likely etiology. The patient’s coagulopathy was rapidly reversed, empiric antibiotic coverage was provided, and she rapidly improved. Brodifacoum exposure has been known to cause increased bleeding, as seen in this case. However, it should also be considered that exposure can lead to epiglottitis. If a similar patient is presented in the future, it is important to consider that coagulopathy may be caused by the adulteration of drugs of abuse, specifically brodifacoum with synthetic cannabinoids.

## Introduction

In March 2018, over 400 people in the United States were poisoned after inhaling synthetic cannabinoids laced with long-acting anticoagulant rodenticides (LAARs). This outbreak caused severe coagulopathy and bleeding in affected patients and resulted in 11 deaths [1,2]. More recently, in December 2021, 50 individuals in Hillsborough County, Florida, were hospitalized with severe coagulopathy and bleeding after using synthetic cannabinoids contaminated with LAAR brodifacoum. This outbreak resulted in four deaths. Our tertiary care center located in Tampa, Florida, along with the Florida Poison Information Center Tampa, treated many of these unique patients. Here, we present a rare case of epiglottitis associated with brodifacoum exposure following synthetic cannabinoid use.

This article was previously presented as a poster at the North American Congress of Clinical Toxicology in San Francisco, California, on September 17, 2022.

## Case presentation

A 33-year-old woman presented to the emergency department with hematuria, vaginal bleeding, and hemorrhagic blebs on her hands, gingiva, and tongue. A computed tomography (CT) scan of the abdomen and pelvis revealed that the patient had a hemorrhagic endometrioma, and the gynecology team was consulted. Labs at the time of admission were significant for an international normalized ratio (INR) >8.5 and a hemoglobin of 11.0 g/dL. She was immediately treated with 10 mg oral vitamin K, followed by 5 mg IV vitamin K. The gynecology team opted for medical management of the patient’s endometrioma given the rapid improvement in her vaginal bleeding, INR, and hemodynamic status after vitamin K administration. Her hemoglobin decreased to 7.6 g/dL, and she received one unit of packed RBCs, from which her hemoglobin (Hgb) improved to 8.9 g/dL. She was also treated with 10 mg IV vitamin K four times a day (QID) for one day while on IV antibiotics, followed by 30 mg oral vitamin K three times a day (TID). Additionally, she was treated with four units of fresh frozen plasma throughout her stay. The patient endorsed synthetic cannabinoid use, and testing for brodifacoum was performed. The patient had an improved INR of 1.4 seven days after admission and left against medical advice on a total daily dose of 50 mg oral vitamin K TID.

Fourteen days after discharge, she re-presented to the emergency department with widespread ecchymosis, left leg swelling, and intermittent gingival and vaginal bleeding. Her lab results again showed an INR > 8.5. The patient reported that she was taking a subtherapeutic 100 mcg twice daily of over-the-counter oral vitamin K after running out of her prescribed vitamin K. She was given 10 mg IV vitamin K, four units of fresh frozen plasma, and one unit of packed RBCs. She was again controlled via 50 mg oral vitamin K TID, stabilized, and the patient again left against medical advice but was given a prescription for 50 mg oral vitamin K TID.

**Figure 1:**
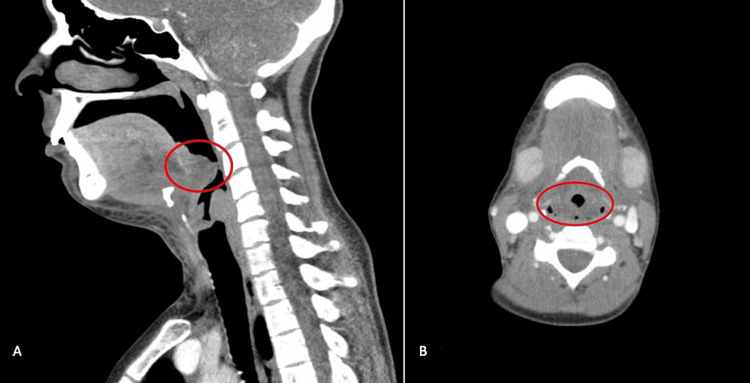
CT soft tissue neck with contrast. (A) Sagittal view. (B) Axial view. Diffuse enlargement edema involving the epiglottis, lingual tonsil, and arytenoids with moderate narrowing of the hypopharynx. CT: computed tomography.

Twenty-eight days following the second discharge, the patient presented to the emergency department for the third time with oral swelling, associated voice changes, right eye ecchymosis, and vaginal bleeding after abstaining from vitamin K therapy for two weeks due to a lack of medication. Her lab results once again showed an INR > 8.5, and a CT soft tissue neck was ordered to help evaluate her complaint of swelling, which demonstrated diffuse enlargement of the epiglottis, arytenoids, and lingual tonsils with a narrowing of the airway at the level of the hypopharynx (Figure 1). Concern was raised for eventual airway compromise and otolaryngology consultation, and a bedside nasopharyngolaryngoscopy showed the base of the tongue, epiglottis, aryepiglottic folds, arytenoids, and false vocal folds were all edematous with diffuse ecchymosis. The patient was intubated with a flexible fiberoptic orotracheal airway intubation to avoid further decompensation. 

The patient received IV and oral vitamin K and was also given empiric antibiotics to cover infectious etiologies, although this was considered less likely due to the absence of fever, leukocytosis, or other infectious features. She was extubated two days later with an INR of 7.2. Subsequently, her INR fully normalized, and she was discharged on day 4 with another prescription of 50 mg oral vitamin K TID.

## Discussion

Epiglottis is most often infectious in nature, but other causes can include thermal exposure to cannabis or crack cocaine, trauma or hematomas associated with hemophilia, and some systemic conditions such as lymphoproliferative disease and chronic granulomatous diseases [3,4]. Although the patient’s epiglottitis could be a result of thermal injury associated with smoking synthetic cannabinoids, given diffuse ecchymosis and severe coagulopathy, hematoma associated with brodifacoum poisoning was considered the most likely etiology.

The standard of care for brodifacoum poisoning is long-term, high-dose oral vitamin K after initial stabilization. Vitamin K therapy is often required for months after exposure due to the long half-life of brodifacoum [5-8]. Clinical recommendations suggest treating with vitamin K until brodifacoum is gradually eliminated to a serum level <10 ng/mL [8]. Adherence to high-dose oral vitamin K was a challenge for our patient, as documented in previous outbreaks [9]. Part of this challenge is the immense cost (~$30,000 USD per month) for cash-paying patients. This raises concern, considering most of the patients impacted by these outbreaks have been medically underserved and lack adequate health insurance. Premature discontinuation of vitamin K1 therapy may lead to a recurrence of coagulopathy and bleeding. We present in this case how a lack of vitamin K1 adherence following brodifacoum exposure can be life-threatening and uniquely present with epiglottitis.

## Conclusions

Brodifacoum exposure in humans has been known to cause coagulopathy and clinical bleeding, as seen in this case. However, clinicians should be aware of brodifacoum and other LAAR exposures’ potential to cause submucosal ecchymosis, bleeding, and edema that affect important anatomical structures, leading to airway compromise. If a patient presents to an emergency department for overdose or ingestion, especially if synthetic cannabinoids are suspected, it is important to consider exposure to LAARs such as brodifacoum and screen for coagulopathy in the workup. Rapid assessment of PT/INR level in patients presenting with substance abuse and bleeding and rapid correction of coagulopathy are critical in preventing life-threatening consequences of exposure to brodifacoum and other LAARs.
